# Virtual Reality and Artificial Intelligence as Clinical Tools in Acute Mental Health: Implementation and Early Outcomes

**DOI:** 10.1192/bjo.2026.11630

**Published:** 2026-06-30

**Authors:** Azmathulla Khan Hameed

**Affiliations:** Cygnet Hospital Harrow, London, United Kingdom

## Abstract

**Aims:**

Acute mental health wards are emotionally intense environments where patients may experience sudden anxiety, distress, agitation, or feeling overwhelmed. In these moments, engagement with verbal therapy can be difficult, and staff often need safe, non-pharmacological options that support rapid emotional regulation while maintaining therapeutic connection.

Virtual Reality (VR), supported by Artificial Intelligence (AI), is increasingly used in healthcare, but it remains uncommon in routine acute mental health practice. This case study describes how an AI-supported VR programme was introduced at Cygnet Harrow as part of standard clinical care and explores feasibility, clinical usefulness, and early outcomes for patients and staff.

**Methods:**

Cygnet Harrow introduced a VR therapeutic programme in partnership with XR Health, using Meta Quest 3 headsets integrated into routine ward care. Importantly, the intervention was delivered as part of Occupational Therapy (OT) activity and therapeutic engagement, rather than as a formal research project.

Staff received structured training covering safe equipment use, patient support, and how to embed VR meaningfully into daily OT-led interventions. Sessions were individualised and voluntary: patients experiencing acute anxiety accessed calming environments (e.g., nature-based, breathing-focused spaces), while those managing agitation accessed grounding-based environments designed to reduce distress and promote emotional regulation.

The VR system generated automated session reports including session duration, engagement indicators, and repeated session patterns, supported by AI-based analytics. Over a six-month implementation period, outcomes were monitored using routinely recorded staff-rated parameters: agitation (0–10), mood distress (0–10), and therapeutic engagement (0–10), alongside qualitative documentation of willingness to participate in wider therapeutic activity.

**Results:**

Eleven patients participated over six months (n=11), completing 94 sessions in total. Attendance was strong, with 82% session completion, and there were no serious adverse events.

Across the cohort, clear improvements were observed. Average agitation scores reduced from 7.1 to 4.2 (mean reduction −2.9 points). Mood distress reduced from 7.8 to 5.0 (mean reduction −2.8 points). Therapeutic engagement increased from 3.6 to 6.4 (mean increase +2.8 points).

In addition 8 out of 11 patients (73%) showed increased willingness to engage in broader therapeutic activity following VR sessions, including 1:1 OT engagement, psychology work, and group interventions. Staff reported VR was particularly useful for patients who were withdrawn, highly distressed, or reluctant to participate through traditional approaches. AI-generated summaries provided objective supporting information that complemented clinical observations and informed MDT decision-making.

**Conclusion:**

AI-supported VR was feasible, safe, and clinically meaningful when delivered as part of routine OT activity in an acute mental health setting. It provided a practical non-pharmacological option for emotional regulation during crisis and supported improved engagement with wider treatment. With continued implementation, this approach may be transferable across other acute services.

